# Task-Related Hemodynamic Changes Induced by High-Definition Transcranial Direct Current Stimulation in Chronic Stroke Patients: An Uncontrolled Pilot fNIRS Study

**DOI:** 10.3390/brainsci12040453

**Published:** 2022-03-28

**Authors:** Heegoo Kim, Jinuk Kim, Gihyoun Lee, Jungsoo Lee, Yun-Hee Kim

**Affiliations:** 1 Department of Physical and Rehabilitation Medicine, Center for Prevention and Rehabilitation, Heart Vascular Stroke Institute, Samsung Medical Center, Sungkyunkwan University School of Medicine, Seoul 06351, Korea; hiheegoo@gmail.com (H.K.); kimjuk92@gmail.com (J.K.); gihyounlee@gmail.com (G.L.); 2 Department of Health Sciences and Technology, SAIHST, Sungkyunkwan University, Seoul 06355, Korea; 3 Department of Medical IT Convergence Engineering, Kumoh National Institute of Technology, Gumi 39253, Korea; 4 Department of Medical Device Management & Research, Department of Digital Health, SAIHST, Sungkyunkwan University, Seoul 06355, Korea

**Keywords:** high-definition transcranial direct stimulation, functional near-infrared spectroscopy, stroke, upper extremity function, oxyhemoglobin concentration

## Abstract

High-definition transcranial direct current stimulation (HD-tDCS) has recently been proposed as a tDCS approach that can be used on a specific cortical region without causing undesirable stimulation effects. In this uncontrolled pilot study, the cortical hemodynamic changes caused by HD-tDCS applied over the ipsilesional motor cortical area were investigated in 26 stroke patients. HD-tDCS using one anodal and four cathodal electrodes at 1 mA was administered for 20 min to C3 or C4 in four daily sessions. Cortical activation was measured as changes in oxyhemoglobin (oxyHb) concentration, as found using a functional near-infrared spectroscopy (fNIRS) system during the finger tapping task (FTT) with the affected hand before and after HD-tDCS. Motor-evoked potential and upper extremity functions were also measured before (T0) and after the intervention (T1). A group statistical parametric mapping analysis showed that the oxyHb concentration increased during the FTT in both the affected and unaffected hemispheres before HD-tDCS. After HD-tDCS, the oxyHb concentration increased only in the affected hemisphere. In a time series analysis, the mean and integral oxyHb concentration during the FTT showed a noticeable decrease in the channel closest to the hand motor hotspot (hMHS) in the affected hemisphere after HD-tDCS compared with before HD-tDCS, in accordance with an improvement in the function of the affected upper extremity. These results suggest that HD-tDCS might be helpful to rebalance interhemispheric cortical activity and to reduce the hemodynamic burden on the affected hemisphere during hand motor tasks. Noticeable changes in the area adjacent to the affected hMHS may imply that personalized HD-tDCS electrode placement is needed to match each patient’s individual hMHS location.

## 1. Introduction

Upper extremity motor impairment is a common sequela after stroke [1,2,3]. Long-term disability of upper extremity motor function in stroke patients causes difficulties in activities of daily living [4,5], returning to work [6,7], social life [8], and quality of life [9,10]. After stroke, performing a task with the affected hand has been shown to increase activity in several cortices within the ipsilesional and contralesional hemispheres to a greater extent than in healthy subjects [11].

Modulation of neuroplasticity is a key factor in the rehabilitation of stroke patients. Transcranial direct current stimulation (tDCS) is a noninvasive brain stimulation technique that can modulate cortical excitability in various ways, depending on the polarity of the induced electrical field (EF) [12]. Thus, it is often used in rehabilitation research to induce neural plasticity [13,14,15]. Conventional tDCS is generally applied using two large (approximately 35 cm^2^) rubber-sponge electrodes. Anodal stimulation with tDCS (1–2 mA) can only increase the rate of spontaneous combustion and their excitability but cannot depolarize the membrane potential of neurons to the firing threshold by itself [16]. On the other hand, cathodal stimulation is thought to deepen the resting membrane potential, making it difficult for neurons to depolarize, which reduces spontaneous combustion rates and the excitability of neurons [16]. By simultaneously applying anodal and cathodal stimulation, while the anode induces neuronal depolarization and thus activation of neural networks beneath the electrode, the cathode induces the opposite effects (i.e., hyperpolarization and consequent inhibition) [17]. Therefore, an anode electrode causes an enhancement of cortical excitability during stimulation, while the cathode electrode generates the opposite effect, i.e., anodal-excitation and cathodal-inhibition effects (AeCi) [18]. Recent tDCS studies have adjusted the size [19], number [20], and placement [21] of electrodes to promote the efficiency of tDCS to the target area.

High-definition transcranial direct current stimulation (HD-tDCS) has recently been developed to increase the spatial precision of current delivery to a target area using arrays of small electrodes [22]. HD-tDCS showed a comparable effect with conventional tDCS on motor learning capacity in healthy children [23], executive function in healthy subjects [24], in tinnitus patients [25], and working memory in children and adolescents with attention deficit hyperactivity disorder [26]. In addition, a previous electroencephalogram (EEG) study demonstrated that the HD-tDCS and anode conventional tDCS are similar in reducing the alpha power in EEG, which induces cortical deactivation and inhibition at resting state in healthy subjects [27]. Using a ring configuration of HD-tDCS electrodes, peak stimulation can be concentrated in a target region [28]. Among the possible arrangements of electrodes for HD-tDCS application, a commonly used configuration is 4 × 1 [29]. In this arrangement, a center ring anodal or cathodal electrode overlying the target cortical regions is surrounded by four cathodal or anodal electrodes depending on the purpose of inducing cortical activity to the target site [30,31]. The ring helps to circumscribe the area of stimulation. A finite element model based on high-resolution magnetic resonance imaging (MRI) predicted that the 4 × 1 ring electrode configuration would focus stimulation compared with a conventional tDCS setup using a rectangular pad [32]. The focality enabled by the HD-tDCS configuration could modulate behavioral and neurophysiological parameters more effectively than conventional tDCS. In previous studies, HD-tDCS has been shown to enhance motor cortex excitability, have longer-lasting effects [33], and improve motor learning capacity [34] compared with conventional tDCS. Additionally, previous HD-tDCS studies demonstrated effects on verbal learning and working memory in healthy subjects and [35] naming in patients with post-stroke aphasia [36], and a decrease in the intrusiveness of tinnitus [37]. A recent EEG study suggested that conventional tDCS and HD-tDCS had different effects in the cortical network during visuomotor processing [38].

Neuroimaging is a methodological approach that can increase understanding of neuronal mechanisms [39]. Functional near-infrared spectroscopy (fNIRS) is a noninvasive optical imaging technique that illustrates cortical activity by quantifying the concentrations of oxyhemoglobin (oxyHb) and deoxyhemoglobin (deoxyHb) using continuous-wave light (650–950 nm) emitted through the skull into the brain [40]. Unlike conventional functional neuroimaging modalities, such as functional MRI (fMRI) and positron emission tomography (PET), fNIRS has a relatively high tolerance to motion artifacts even during motor tasks [40,41]. Furthermore, fNIRS imaging can detect continuous hemodynamic variation in everyday life situations in a cost-effective and portable manner [42]. Therefore, the use of fNIRS in clinical trials is expanding [43,44,45].

Recent fNIRS studies of HD-tDCS unveiled the hemodynamic correlate of a 4 × 1 HD-tDCS electric field on the brain and demonstrated changes in neuroplasticity [46,47]. Another fNIRS study suggested that the functional connectivity of the dorsolateral prefrontal cortex increased after HD-tDCS in healthy subjects [48]. Furthermore, an fNIRS study as well as behavioral studies on the effect of focal stimulation of HD-tDCS on upper limb motor function in stroke patients have been proposed [49].

Therefore, we aimed to collect preliminary evidence on hemodynamic changes and cortical activation in stroke patients by applying HD-tDCS with a 4 × 1 ring electrode configuration to their motor areas. We used fNIRS to investigate interhemispheric cortical excitability and changes in oxyHb concentration in chronic stroke patients during a hand motor task before and after an HD-tDCS intervention. As a pilot investigation, we hypothesized that applying 4 × 1 HD-tDCS to the motor areas of stroke patients would modulate the interhemispheric imbalance found during a hand motor task after stroke to a more normal interhemispheric interaction and lower the cortical activity required to perform the hand motor task. We further hypothesized that this effect would be more pronounced in the cortical area related to hand motor function.

## 2. Materials and Methods

### 2.1. Participants

We enrolled 30 participants in this uncontrolled pilot study, but 4 (13%) of them withdrew their consent prior to the intervention. Thus, 26 chronic stroke patients (20 males and 6 females, mean age 59.4 ± 12.8 years) completed this study. The inclusion criteria were as follows: unilateral hemiparetic stroke, age between 19 and 80 years, chronic strokes for more than 6 months, subcortical lesion stroke, and ability to move individual fingers. The exclusion criteria were history of psychiatric disease, significant neurological disease other than stroke, metal implants, and contraindications to tDCS application [50]. The patient demographics are summarized in Table 1. All participants provided written informed consent before participation. The experimental procedures were approved by the Ethics Committee of Samsung Medical Center. This study was registered at ClinicalTrials.gov (NCT0459753).

### 2.2. Study Design

**Figure 1:**
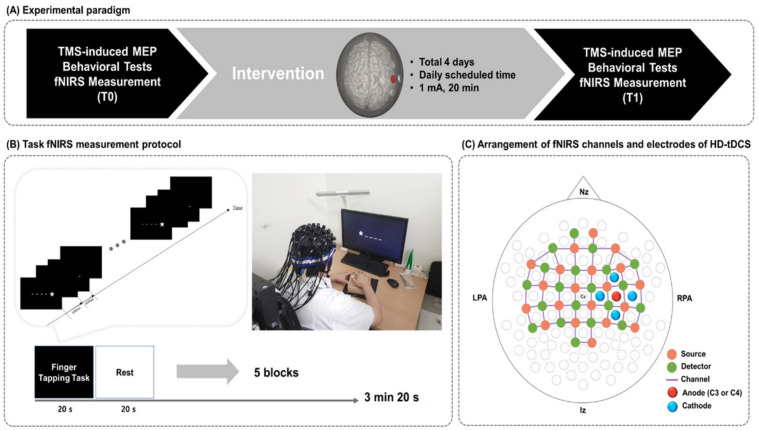
Study design. (**A**) Experimental paradigm. (**B**) fNIRS measurement during the FTT. A star appeared on the black screen for 600 ms, and then an empty black screen appeared for 400 ms after the star disappeared. Each subject pushed the corresponding buttons using fingers on the affected side. (**C**) Arrangement of fNIRS optodes and HD-tDCS electrodes. fNIRS, functional near-infrared spectroscopy; FTT, finger tapping task; HD-tDCS, high-definition transcranial direct current stimulation; Nz, nasion; Iz, inion; LPA, left pre-auricular; RPA, right pre-auricular.

Using an open-label, single-arm, uncontrolled pilot study design, all participants completed four consecutive daily sessions of HD-tDCS at daily scheduled time. To measure hemodynamic changes, fNIRS was conducted during the finger tapping task (FTT) before (T0) and immediately after (T1) the HD-tDCS intervention. In addition, to examine the corticomotor excitability, the resting motor threshold (rMT) and amplitude of the motor evoked potential (MEP) were evaluated at T0 and TMotor function of the affected hand was assessed at the same time points using the Fugl-Meyer assessment (FMA), box and block test (BBT), and FTT accuracy and response time. The study design is illustrated in Figure 1.

### 2.3. High-Definition tDCS

A battery-driven Starstim 8 tDCS system (Neuroelectrics^®^, Barcelona, Spain) was used to deliver constant direct current to the affected hemisphere via a 4 × 1 ring montage of HD electrodes (surface: 3.14 cm^2^; current density: 0.32 mA/cm^2^). The anode was placed on the scalp overlying C3 or C4 (based on the 10–20 system) to cover the ipsilesional motor cortical area. The four cathodes surrounded the anode at a center-to-center distance of 3.5 cm. Thus, when a participant’s lesion was on the left side, the anode was placed on C3, and the cathodes were placed on C1, C5, FC3, and CPWhen, on the other hand, a participant’s lesion was on the right side, the anode was placed on C4, and the cathodes were placed on C2, C6, FC4, and CPConstant current was delivered at 1 mA for 20 min, with ramp-up and -down phases of 30 s.

### 2.4. Measurement of Hemodynamic Changes during the Finger Tapping Task

Hemodynamic changes during the FTT with the affected hand were measured in each patient at T0 and TThe hemodynamic change signals were obtained as optical changes collected by a continuous wave fNIRS measurement system (NIRScout^®^; NIRx Medical Technology, Berlin, Germany), which is a multi-modal-compatible fNIRS platform. The fNIRS system used two wavelengths, 760 nm and 850 nm, with the sampling rate set to 10.25 Hz. Using 20 sources and detectors, the fNIRS topomap consisted of 67 channels with a distance of 3 cm between each source and detector. The fNIRS topomap covered the frontal, parietal, temporal, and occipital cortices. During the fNIRS measurements, all patients performed the FTT with the affected hand. The acquisition software NIRStar 15.2 (NIRx Medical Technologies, Berlin, Germany) was used to record the raw fNIRS data and obtain signal quality indicators for the measurement channels following hardware calibration. If the acquired signal quality was poor during calibration, the contact between the scalp and analogous optodes was immediately adjusted until the overall signal quality was acceptable. An FTT protocol programmed using SuperLabPro^®^ 2.0 software (Cedrus, Co., Phoenix, AZ, USA) was conducted for all participants (Figure 1). It consisted of random-ordered sequences of five task and rest blocks, each lasting for 20 s.

During the FTT with fNIRS measurement, each patient was seated 50 cm from a computer monitor, and the affected hand performing the task was held in a supported position. As a visual cue on the monitor, one star randomly appeared at one of five positions arranged in a horizontal line in front of the patient. The patient was asked to press a button corresponding to a stimulus presented on the screen with their affected fingers as quickly and accurately as possible when a star appeared at a specific location (thumb = 1, index finger = 2, middle finger = 3, ring finger = 4, little finger = 5). A star appeared for 600 ms, after which a black screen appeared on the monitor for 400 ms. Random-ordered sequences were assigned for each patient at T0 and T1.

### 2.5. fNIRS Data Anlysis

The cortical activation map produced during the FTT with the affected hand was analyzed using statistical parametric mapping (SPM) analysis with the Near-Infrared Spectroscopy-Statistical Parametric Mapping open-source software package (NIRS-SPM; http://bisp.kaist.ac.kr/NIRS-SPM, accessed on 3 February 2021) [51] implemented in a MATLAB^®^ environment (MathWorks, Inc., Natick, MA, USA). A general linear model with a canonical hemodynamic response curve was used to test for significant changes in oxyHb concentration during task periods compared with rest periods [52]. The group-level statistical analysis was performed based on the individual-level beta values to detect activated channels at the group level (*p* < 0.05, uncorrected) [53]. Group-level cortical activation maps were plotted onto a standard brain template with flipped channels to align the affected hemisphere, and the regions with significant differences in oxyHb concentration were identified.

Changes in oxyHb and deoxyHb concentrations were analyzed using nirsLAB^®^ software (v. 2019.04; NIRx Medical Technologies, LLC, Minneapolis, MN, USA) for a time series analysis. Discontinuities and spike artifacts acquired from 67 channels were removed and replaced by the nearest signals. First, the raw data were band-pass filtered from 0.01 to 0.2 Hz to remove baseline noise and to eliminate possible respiration and heart rate signals [54]. The band-pass filter is a combination of a low-pass and high-pass filter, in that it passes a certain band of frequencies and attenuates the frequencies located outside the band [55]. Second, the oxyHb and deoxyHb concentrations were calculated from the preprocessed and filtered data using the Beer–Lambert law for each of the 67 channels [56], and the grand average of the hemodynamic response in each channel was computed. Both the mean and integral values of oxyHb and deoxyHb concentration changes were obtained during each 20-s task block from the channels around the tDCS stimulation for comparison between T0 and T1.

### 2.6. Identification of the Hand Motor Hotspot and Motor Evoked Potential Study

To measure changes in corticospinal excitability at T1 compared with T0, single-pulse transcranial magnetic stimulation (TMS) was performed at T0 and TWe used a TMS system (Magstim^®^ BiStim^2^; Magstim Co. Ltd., Dyfed, Wales, UK) and a 70-mm figure-eight coil. First, electromyography (EMG) data were acquired from the contralateral first dorsal interosseus muscle based on a muscle belly tendon montage using a self-adhesive surface electrode. An EMG monitoring system (Medelec Synergy^®^; Medelec, Oxford, UK) was used to amplify the EMG activity, and the data were band-pass filtered from 10–2000 kHz. Second, the vertex (Cz) and ipsilesional C3 or C4 points were marked based on the international 10–20 system. Third, the examiner oriented the handle of the coil 45° posterior to the midline to ensure that the electromagnetic current was transmitted perpendicular to the central sulcus. In the previous studies, C3 or C4 based on the 10–20 system is not always consistent with the TMS-induced hand motor hotspot (hMHS) [57,58]. Therefore, we determined the location of hMHS where the optimal location exerted the highest MEP amplitude and the shortest latency by moving 1 cm in each direction at 5-s intervals around the ipsilesional C3 or CThen, we recorded the location hMHS in both hemispheres based on the distance from Cz to the x and y axes in each participant.

After the hMHS was identified, single-pulse TMS was gradually delivered to define the overlying rMT, defined as the lowest magnetic intensity that induced EMG activity (MEP peak-to-peak amplitude ≥50 μV) in 5 or more of 10 consecutive trials. Following rMT determination, the MEP amplitude was calculated as the average amplitude obtained by 10 single hMHS stimuli 5 s apart at an intensity of 120% rMT. To assess relaxation of the measured muscle, the examiner carefully monitored real-time EMG before stimulation [59]. During the examination, the participant sat in a comfortable recliner and held their hands in a supine position on their lap while the measurement was performed. Participants were asked to remain silent during the experiment to prevent speech-induced modulation of cortical excitability. The identification of hMHS and measurements of rMT and MEP amplitude were performed in both affected and unaffected hemispheres.

### 2.7. Behavioral Assessments

To assess functional changes in the affected upper extremity, the patients completed a battery of behavioral assessments at T0 and T1, and the FTT accuracy and response time were used to assess upper extremity function. The FMA is a comprehensive quantitative measurement of sensorimotor impairment after stroke [60]. The FMA motor assessments for the upper (maximum score 66 points) and lower (maximum score 34 points) extremity are recommended as core measures to be used in every stroke recovery and rehabilitation trial [61]. The BBT was used to assess gross manual dexterity with a wooden box divided into two equal compartments by a partition and 150 blocks. With the box oriented lengthwise and placed at the patient’s midline, the examiner asks the patient to move as many blocks as possible, one by one, from one compartment to the other within 60 s [62].

To measure FTT performance, each patient’s mean response time and number of correct responses (accuracy) were calculated with SuperLabPro^®^ software. The response time was defined as the mean time required for the patient to press the correct key after appearance of the stimulus on the screen. The accuracy and response time were measured for 20 stimuli within each trial, with five trial blocks for each task.

### 2.8. Statistical Analysis

The data were analyzed using SPSS version 20 (SPSS, Inc., Chicago, IL, USA). To evaluate the normality of the distribution, the data were examined using the Kolmogorov–Smirnov test, and the mean and integral values of the oxyHb and deoxyHb concentrations in each channel were found to have nonparametric distributions. The Wilcoxon signed-rank test was used to confirm the statistical significance of the mean and integral values of the oxyHb and deoxyHb concentrations in each channel at T0 and TDue to using the Wilcoxon signed-rank test for oxyHb and deoxyHb concentrations, we calculated the effect size using the following formula (Equation (1)) [63]:

(1) $$r=\frac{Z}{\sqrt{N}}$$

*Z* represents the z-statistics from the Wilcoxon signed-rank test, and *N* represents the number of participants. All of the neurophysiologic and behavioral assessment variables showed parametric distributions. Therefore, paired *t*-tests were used to compare the neurophysiological measurements and behavioral assessments at T0 and TDue to using paired *t*-tests for the neurophysiologic and behavioral assessments, we calculated the effect sizes using the following formula (Equation (2)) [63]:

(2) $$d=\frac{mean_{D}}{SD_{D}}$$

$mean_{D}$ represents the mean difference between T0 and T1, and $SD_{D}$ represents the mean of the standard deviation between T0 and TFor all analyses, the level of significance was set at *p* = 0.05.

## 3. Results

### 3.1. Cortical Hemodynamic Changes during Finger Tapping Task

**Figure 2:**
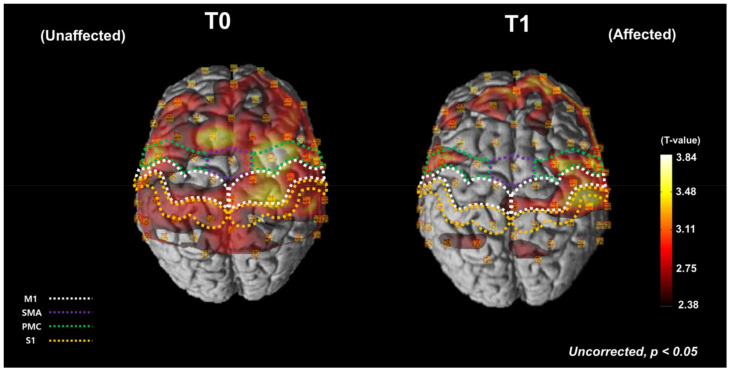
Average cortical activation maps, as analyzed using the NIRS-SPM software during the FTT with the affected hand before and after HD-tDCS intervention. The white dotted areas indicate the MThe green dotted areas indicate the SMA. The purple dotted areas indicate the PMC. The orange dotted areas indicate the SAt T0, the cortical oxyHb concentration increased during the FTT in both the affected and unaffected hemispheres. At T1, the overall cortical activation was decreased and most of the activation was shifted to the affected hemisphere. FTT, finger tapping task; T0, before the intervention; T1, after the intervention; M1, primary motor cortex; SMA, supplementary motor area; PMC, premotor cortex; S1, primary somatosensory cortex; oxyHb, oxyhemoglobin.

Figure 2 shows the average cortical activation during the FTT with the affected hand at T0 and T1, as shown by the NIRS-SPM analysis. During the FTT before the HD-tDCS intervention, cortical activation increased in both affected and unaffected hemispheres, especially around the central areas of the affected hemisphere (Figure 2, left). After the intervention, overall cortical activation decreased, and most of the activation shifted to the affected hemisphere (Figure 2, right).

**Figure 3:**
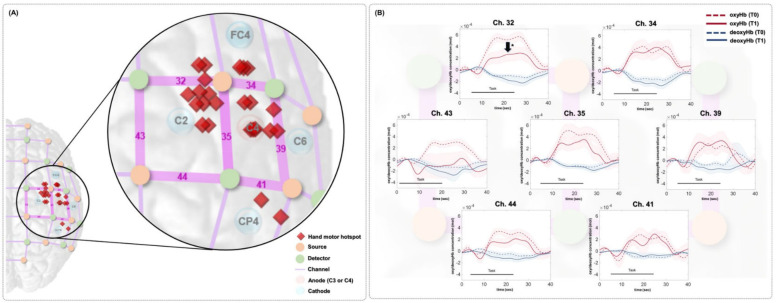
(**A**) Location of the fNIRS channels. The red rhombi represent the individual hMHS locations. The anode electrode was placed on the ipsilesional hemisphere of each participant (C3 or C4). When the anode was on C3, the cathodes were placed on C1, C5, FC3, and CPWhen the anode was on C4, the cathodes were placed on C2, C6, FC4, and CPIn this figure, all patients were assumed to have the right-sided lesions, so the location of the fNIRS channels, optodes, HD-tDCS electrodes, and individual hMHS locations are expressed in the right hemisphere. (**B**) Results of time series oxyHb concentration changes in the affected motor area in each fNIRS channel during the FTT. The red dotted and solid lines represent the oxyHb concentration at T0 and T1, respectively. The blue dotted and solid lines represent the deoxyHb concentration at T0 and T1, respectively. The colored background represents the standard error. In channel 32, the oxyHb concentration was significantly decreased at T1 compared with T0. hMHS, hand motor hotspot; oxyHb, oxyhemoglobin; deoxyHb, deoxyhemoglobin; T0, before intervention; T1, after intervention; FTT, finger tapping task.

Figure 3 shows locations of the fNIRS optodes and channels and the arrangement of the HD-tDCS electrodes (Figure 3A). The time series data for the oxyHb and deoxyHb concentrations around the stimulation site during the FTT are presented in Figure 3B. In channels 32, 35, 43, and 44, the oxyHb concentration decreased during the FTT at T1 compared with TThe mean and integral values of oxyHb tended to decrease after the HD-tDCS intervention in all four of those channels, and statistically significant decreases in the mean and integral values of the oxyHb concentration were observed at T1 compared with T0 in channel 32 (*p* < 0.05; Table 2). There were no significant changes in both mean and integral values of the deoxyHb concentration in the channels of the stimulated site at T1 compared with T0 in all analyzed channels (Table 2). Most of the hMHSs (16 of 24 participants) were located anterior or medial to the stimulation site (C3 or C4), and the hMHS in nine participants was located close to channel In the other channels, the mean and integral oxyHb values tended to decrease after the intervention compared with the values before the intervention, but the differences were not statistically significant.

### 3.2. Changes in Behavioral Test Results and Corticospinal Excitability Measurement

The FMA upper extremity scores improved significantly after the intervention (*p* < 0.001). Both the FMA upper extremity mean score and FMA total score were significantly higher at T1 than at T0 (*p* < 0.001). The BBT score also increased significantly after the HD-tDCS intervention (*p* = 0.001). Furthermore, FTT accuracy improved significantly, by 35.47%, after the intervention (T1) compared with T0 (*p* = 0.001). The FTT response time tended to decrease at T1 compared with T0, but that difference was not statistically significant (*p* > 0.05).

In the TMS-induced MEPs in the affected hemisphere, rMT decreased slightly but without statistical significance at T1 compared with T0 (*p* > 0.05). The MEP amplitude in the affected hemisphere tended to increase slightly at T1, but that difference was also without statistical significance (*p* > 0.05; Table 3).

## 4. Discussion

In this uncontrolled pilot study, we investigated changes in the cortical hemodynamic response after HD-tDCS of the ipsilesional motor cortical area in chronic stroke patients to guide the implementation of future controlled studies. The HD-tDCS intervention could modulate the cortical oxyHb concentration changes toward an overall decrease in bilateral hemispheric activation and focused activation in the affected motor cortical areas, in accordance with improved functional performance of the affected hand. In addition, a pronounced decrease in task-related cortical activation of the affected motor cortical area was evident at the channel closest to the hMHS.

Before the HD-tDCS intervention, we observed overall cortical activation in both the affected and unaffected hemispheres of stroke patients during the FTT. This abnormal interhemispheric pattern is related to disruption of interhemispheric inhibitory balance caused by stroke [64,65]. Conventional tDCS studies have suggested that interhemispheric imbalance could be decreased by properly placing anode and cathode electrodes on the affected and unaffected hemispheres, respectively [66,67]. A previous fNIRS study in healthy subjects demonstrated increased interhemispheric connectivity after applying HD-tDCS to the dorsolateral prefrontal cortex [48]. In addition, Cabibel et al. found that applying HD-tDCS to upper extremity cortical hotspots can enhance cross-facilitation, increasing the excitability of unstimulated areas [68]. After the HD-tDCS intervention in this uncontrolled pilot study, cortical activation appeared predominantly in the affected hemisphere, and the overall activity in the unaffected hemisphere decreased. This cortical activation was similar to the asymmetric cortical activation seen in healthy subjects with normal interhemispheric inhibitory balance [69]. This result might imply that HD-tDCS can induce rebalancing of interhemispheric inhibition caused by stroke.

Our time series analysis showed that, after the HD-tDCS intervention, the oxyHb concentration decreased in the affected motor area during the FTT compared with before the intervention. Although the changes of deoxyHb between T0 and T1 showed a similar tendency to the changes of oxyHb, there were no significant changes in both mean and integral values of the deoxyHb between T0 and TThis might be reflected in that deoxyHb showed an inferior signal-to-noise ratio (SNR) relative to oxyHb [70]. At the same time, the hand motor function of the participants improved after the HD-tDCS intervention. Any increase or decrease in cortical activation required for motor tasks by stroke patients indicates changes in the neural resources required to achieve certain movements [71]. Therefore, decreased oxyHb concentration required for the FTT after HD-tDCS intervention might be interpreted as decreased hemodynamic burden (i.e., neural resources) needed to successfully perform the FTT. Based on previous studies, a decrease in the cortical activation required for a task in stroke patients reflects neuroplastic changes caused by therapeutic intervention [43,71,72,73]. Our result might provide evidence that HD-tDCS can modulate such neuroplastic changes and improve neural efficiency by enabling lower cortical activation to generate better function [74].

In our uncontrolled pilot study, the oxyHb concentration during the hand motor task was decreased in the channels of the affected motor areas. Specifically, the task-related hemodynamic change induced by HD-tDCS was apparent in the fNIRS channel corresponding to the hMHS of most participants. The hMHS could thus be regarded as the best location for tDCS intervention to show changes in the task-related hemodynamic response. The hMHS is the scalp position at which TMS generates the largest MEPs in the hand muscles [75]. According to previous EEG studies, hMHS locations were adjacent to the EEG channel locations that well reflect hand movements [76,77]. Previous PET [78] and fMRI [79] studies demonstrated that both the hMHS and the area of maximal cerebral activation were located in the anatomical hand knob. Therefore, the hMHS might be considered one of the HD-tDCS target sites to effectively modulate cortical excitability related to hand motor function. In the HD-tDCS using a 4 × 1 ring electrode configuration, focality is accompanied with interindividual variability of EF [80]. Therefore, our result that hemodynamic change induced by HD-tDCS with a 4 × 1 ring electrode configuration prominently observed in the fNIRS channel near the hMHS of most participants might propose a considerate placement of HD-tDCS electrodes with a 4 × 1 ring electrode configuration. The location of the hMHS reflects the neurophysiological features of motor cortex excitability and can vary by individual [81,82,83]; personalized HD-tDCS electrode placement considering these features will be required in the application of HD-tDCS using a 4 × 1 ring electrode configuration.

In the behavioral results, functional performance improved significantly after HD-tDCS on the ipsilesional C3 or CThe FMA upper extremity scores, which reflect the overall function of the upper extremity in stroke patients, improved after the intervention, as did the BBT scores and FTT accuracy and response time, which reflect gross hand function and hand dexterity, respectively. Therefore, repeated HD-tDCS application could modulate functional performance in accordance with hemodynamic changes in the relevant cortical areas. In contrast to a previous HD-tDCS study of healthy subjects [33], we did not observe significant differences in neurophysiological responses, represented by rMT and MEP amplitude, even though we applied HD-tDCS with the same current intensity as used in those healthy subjects. Corticomotor excitability in stroke patients might respond to HD-tDCS differently than that in healthy subjects, but that possibility needs further study.

Our uncontrolled pilot study had several limitations. The main limitation was its open-label nature, and there was no control condition using sham or conventional tDCS to compare the effect of real HD-tDCS. Therefore, our preliminary data showing hemodynamic changes induced by HD-tDCS in stroke patients certainly propose the necessity of future confirmatory studies with randomized controlled trials. Second, our four HD-tDCS treatments were not enough to verify the residual effect of HD-tDCS. Third, no changes in cortical hemodynamic responses during HD-tDCS could be identified through fNIRS measurements. Fourth, because the statistical power was relatively low due to our small sample size, our results cannot be generalized to a wider stroke population. Therefore, future research should be performed using a larger sample and more intervention sessions to demonstrate the clinical efficacy of HD-tDCS after stroke. Finally, the recorded fNIRS signals reflect both extra-brain and intra-brain changes. Several of the issues mentioned with fNIRS signals are limitations of our uncontrolled pilot study. The acquisition of fNIRS signals with additional systemic physiological sensors has to be considered in future studies.

## 5. Conclusions

The present uncontrolled pilot study provided some evidence that HD-tDCS intervention could change task-related hemodynamic responses and could help in the rebalancing of bilateral cortical activity in chronic stroke patients. Our results of preliminary data showed that HD-tDCS intervention also could reduce the hand-motor-task-related hemodynamic burden on the affected hemisphere. The hemodynamic change induced by HD-tDCS was most apparent in the fNIRS channel corresponding to the hMHS location in most participants. These results might imply the need to personalize HD-tDCS electrode positioning based on individual neurophysiological studies to improve the effectiveness of the HD-tDCS intervention. An exploratory randomized controlled trial is warranted to verify the preliminary evidence of HD-tDCS.

## Figures and Tables

**Table 1:** Basic patient characteristics.

Characteristics	Value
Age, years (mean ± SD)	59.4 ± 12.8
Sex (Male:Female)	20:6
Stroke type (Infarction:Hemorrhage)	13:13
Lesion side (Left:Right)	12:14
Duration, months (mean ± SD)	40.1 ± 29.4
Initial FMA upper extremity score (mean ± SD)	47.6 ± 10.2

SD, standard deviation; FMA, Fugl-Meyer assessment.

**Table 2:** Changes in mean and integral values of oxyHb and deoxyHb in the channels of motor cortical areas in the affected hemisphere during FTT.

	Mean Value (Units: mol × 10^−3^)				Integral Value (Units: mol × 10^−3^)			
	T0	T1	*p*-Value	Effect Size	T0	T1	*p*-Value	Effect Size
oxyHb								
Ch. 32	0.324 (0.134)	0.157 (0.674)	0.033 *	−0.321	67.07 (63.91)	32.46 (69.37)	0.033 *	−0.321
Ch. 34	0.275 (0.300)	0.265 (0.295)	0.570	−0.086	57.13 (62.12)	54.97 (61.13)	0.570	−0.086
Ch. 35	0.306 (0.367)	0.244 (0.412)	0.445	−0.115	63.45 (76.09)	50.58 (85.39)	0.445	−0.115
Ch. 39	0.183 (0.280)	0.020 (0.351)	0.733	−0.051	38.01 (58.06)	41.63 (72.84)	0.733	−0.051
Ch. 41	0.130 (0.386)	0.154 (0.476)	0.592	−0.081	27.00 (80.09)	32.05 (98.65)	0.592	−0.081
Ch. 43	0.137 (0.169)	0.027 (0.384)	0.088	−0.257	28.53 (35.02)	55.46 (79.42)	0.088	−0.257
Ch. 44	0.181 (0.195)	0.093 (0.216)	0.062	−0.281	37.52 (40.40)	19.16 (44.68)	0.062	−0.281
deoxyHb								
Ch. 32	−0.429 (−0.927)	−0.509 (−1.420)	0.858	−0.027	−8.889 (−19.206)	−10.533 (−29.428)	0.858	−0.027
Ch. 34	−0.609 (−1.146)	−0.893 (−1.789)	0.115	−0.237	−12.589 (−23.740)	−18.495 (−37.060)	0.115	−0.237
Ch. 35	−0.732 (−1.306)	−0.532 (−1.244)	0.910	−0.017	−15.175 (−27.041)	−11.011 (−25.757)	0.910	−0.017
Ch. 39	−0.352 (−0.703)	−0.505 (−0.748)	0.189	−0.198	−7.289 (−14.552)	−10.450 (−15.496)	0.189	−0.198
Ch. 41	−0.292 (−1.021)	−0.226 (−0.574)	0.291	−0.159	−6.056 (−21.140)	−4.668 (−11.889)	0.291	−0.159
Ch. 43	−0.475 (−1.016)	−0.754 (−1.201)	0.465	−0.110	−9.858 (−21.050)	−15.610 (−24.840)	0.465	−0.110
Ch. 44	−0.280 (−0.797)	−0.652 (−1.074)	0.149	−0.218	−5.792 (−16.481)	−13.558 (−22.251)	0.149	−0.218

All data are expressed as median (interquartile range). oxyHb, oxyhemoglobin; deoxyHb, deoxyhemoglobin; FTT, finger tapping task; T0, before the intervention; T1, immediately after the intervention. * Wilcoxon signed-rank test, *p* < 0.05.

**Table 3:** Changes in behavioral test and neurophysiological measurement results.

	T0	T1	*p*-Value	Effect Size
FMA upper extremity (score)	47.6 (10.2)	50.6 (10.3)	<0.001 *	1.308
FMA total (score)	69.3 (14.1)	73.7 (14.4)	<0.001 *	1.009
BBT (ea)	30.0 (16.8)	32.6 (17.4)	0.001 *	0.648
FTT accuracy (%)	33.6 (22.3)	45.7 (27.0)	0.001 *	0.777
FTT response time (ms)	589.1 (106.4)	575.3 (101.8)	0.062	−0.117
rMT of affected hemisphere (%)	51.6 (11.6)	50.83 (9.7)	0.259	−0.231
MEP amplitude of affected hemisphere (μV)	430.1 (313.8)	434.8 (363.7)	0.665	0.088
rMT of unaffected hemisphere (%)	48.6 (9.6)	46.8 (9.3)	0.102	−0.332
MEP amplitude of unaffected hemisphere (μV)	612.9 (306.4)	734.3 (378.0)	0.120	0.316

All data are expressed as mean (standard deviation). T0, before the intervention; T1, immediately after the intervention; FMA, Fugl-Meyer assessment; BBT, box and block test; FTT, finger tapping task; MEP, motor evoked potential; rMT, resting motor threshold. * Paired *t*-test, *p* < 0.05.

## Data Availability

The data that support the findings of this study are available from the corresponding author upon reasonable request.
